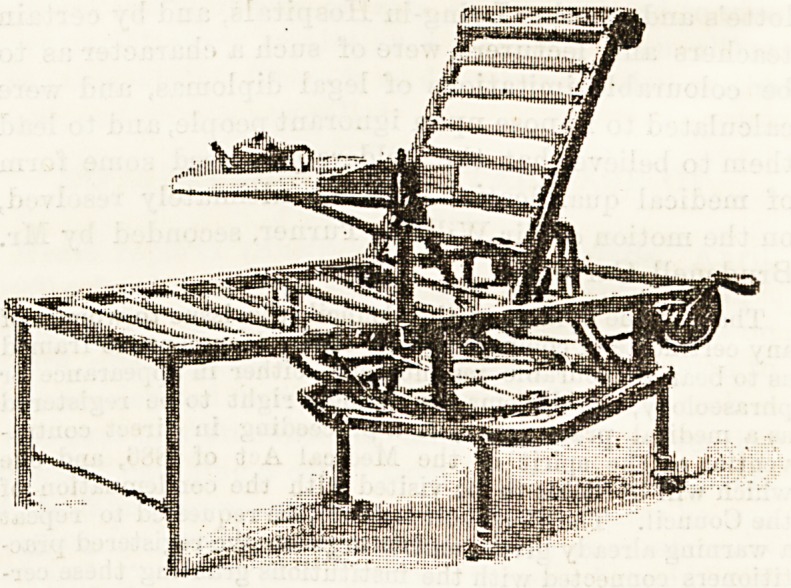# Practical Departments

**Published:** 1894-06-02

**Authors:** 


### PRACTICAL DEPARTMENTS.

### RUSH GAUNTLETS.

We have received some gauntlets made of rush from Messrs. W. B. Fordham and Sons, York Road, King's Cross, N., which will be useful as a sleeve protection for nurses and others. White cuffs soil all too easily, and for temporary use these gauntlets will be just the thing, slipping easily over sleeve and cuff. This illustration is given by kind permission of Messrs. Fordham.

### CHAIR BEDSTEAD.

A useful and convenient chair bedstead has been invented and patented by Mr. Thomas Turner, of Ketton, Stamford. The accompanying illustration gives a good idea of its special features, one of which is its being made to turn on a circular frame so that the patient can be moved round in any direction quite easily without the exercise of such physical force as would be required to shift the position of the whole bedstead. The head and foot parts are controlled by a winding arrangement, which enables them to be raised or lowered as desired. The head mattress can be fixed to prevent that slipping sensation which is so uncomfortable for a sick person. The chair can be also raised to allow of a bed-pan being used if required. We should not omit to mention the happy arrangement of a revolving tray, attached by a mechanical contrivance to the side of the chair, most convenient for tea-tray or book. This chair bedstead will be found very useful in the sick room and for convalescents in hospital wards, where the luxury of a comfortable chair or couch is so immensely appreciated. There is much room for charitable gifts of this kind in hospitals and convalescent homes, and if those who have easy chairs and couches in abundance would visit a hospital ward and look with a seeing eye at the many invalids, up for the first time, with, perhaps, nothing more reposeful to rest on than a hard, straight-backed chair, and remember their own past days of convalescence, hearts and purses would surely be more widely opened to provide this much of comfort for those invalids whose days "in hospital" are often but a brief respite from a toiling life.

## Figures and Tables

**Figure f1:**
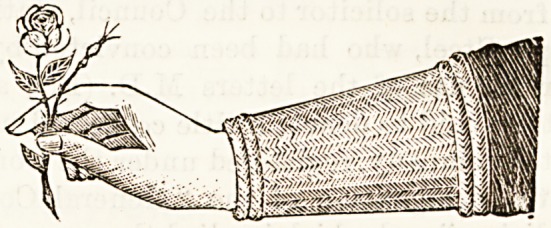


**Figure f2:**